# Pyridine‐Regulated Lamellar Nickel‐Based Metal–Organic Framework (Ni‐MOF) for Nonenzymatic Electrochemical Glucose Sensor

**DOI:** 10.1002/advs.202304102

**Published:** 2023-07-20

**Authors:** Qian Zhang, Panpan Li, Jun Wu, Yi Peng, Huan Pang

**Affiliations:** ^1^ School of Chemistry and Chemical Engineering Yangzhou University Yangzhou 225009 P. R. China

**Keywords:** metal–organic frameworks, morphological control, nonenzymatic electrochemical glucose sensor

## Abstract

2D metal–organic frameworks (MOFs) are considered as promising electrochemical sensing materials and have attracted a lot of attention in recent years. Compared with bulk MOFs, the construction of 2D MOFs can increase the exposure of active sites by obtaining a larger surface area ratio. Herein, a facile one‐pot hydrothermal synthesis of pyridine‐regulated lamellar Ni‐MOFs with ultrathin and well‐defined 2D morphology is described. Compared with the bulk structure, the 2D lamellar Ni‐MOF has higher surface area and active site density, showing better electrochemical glucose sensing performance. The 2D lamellar Ni‐MOF exhibits a fast amperometric response of less than 3 s and a high sensitivity of 907.54 µA mm ^−1^ cm^−2^ toward glucose with a wide linear range of 0.5–2665.5 µm. Furthermore, the 2D lamellar Ni‐MOF also possesses excellent stability and reproducibility, and can be used to detect glucose with high accuracy and reliability in different environments.

## Graphical abstract

2D metal–organic frameworks (MOFs) have been the most interesting materials in recent years. Compared with bulk materials, the construction of 2D MOFs can also increase the exposure of active sites by obtaining a larger surface area ratio. Here, a 2D lamellar Ni‐MOF for nonenzymatic electrochemical glucose sensor is developed.

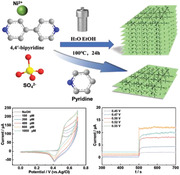

## 1 Introduction

The detection of glucose (Glu) in the food, beverage, fermentation manufacturing sector, and even the medical sector has great significance and market demand.^[^ ^1^ ^]^ Increased sugar intake in people's diets has been linked to a number of chronic health problems, particularly diabetes, which leads to metabolic disorders and even death.^[^ ^2^ ^]^ Meanwhile, numerous studies on the management of diabetes have shown that rigorous blood Glu monitoring can significantly reduce diabetes‐related mortality by delaying the onset and progression of diabetes‐related complications.^[^ ^3^ ^]^ Therefore, people with diabetes need to accurately determine their blood Glu levels not only at the diagnostic stage, but also at all stages of treatment and disease management, and the use of noninvasive, rapid blood Glu level detection methods is essential.^[^ ^4^ ^]^

A variety of Glu detection devices have been invented in the past decades (e.g., electrical, thermal, photoelectric sensors).^[^ ^5^ ^]^ Among these detection devices, electrochemical detection devices have attracted much attention due to their excellent features of simplicity of operator, good sensitivity, and low cost.^[^ ^6^ ^]^ Electrochemical Glu sensors can generally be divided into two categories: enzyme‐based sensors and nonenzyme‐based sensors. The former deliver high specificity, sensitivity, and wide response range.^[^ ^7^ ^]^ However, due to the inherent instability of the enzyme molecule, they are sensitive to changes in environmental conditions (e.g., temperature, humidity, and pH).^[^ ^8^ ^]^ In addition, enzymes also have the characteristics of high cost and difficult preservation, which limit the development of enzyme‐based electrochemical Glu sensors. Therefore, nonenzymatic Glu sensors, i.e., the determination of Glu concentration by electrochemical amperometry in the absence of enzymes, are highly desirable because they can directly oxidize Glu molecules on the electrode surface and can overcome the shortcomings of enzyme‐based Glu sensors.^[^ ^9^ ^]^ Noble‐metal‐based electrodes have a high catalytic activity against Glu, but their easy inactivation and expensive materials limit their commercial applications.^[^ ^10^ ^]^ Therefore, it is necessary to develop a low‐cost electrode material with high sensitivity.^[^ ^11^ ^]^

Metal–organic frameworks (MOFs), which consist of metal ion centers combined with organic linkers, have applications in photo‐/electrocatalysis, gas separation, batteries, drug delivery, and electrochemical sensors on account of their highly diverse structure, adjustable pore structure, high surface area, and customizable chemical properties.^[^ ^12^ ^]^ Early studies in these areas focused on bulk MOFs and found that the properties of materials are limited by the slow mass and charge transfer processes caused by their form and size, so it is necessary to take appropriate measures to regulate the form and size of MOFs.

For electrochemical‐related research, the larger the specific surface area of the materials, the more active sites there are, which is conducive to improved catalytic sensing efficiency. Compared to conventional bulk MOFs, 2D MOFs have a larger specific surface area and more active sites, making them more desirable electrode materials.^[^ ^13^ ^]^ The low thickness of 2D MOFs can also shorten the mass transfer and charge transfer pathways and enrich the active sites on the surface. The top‐down and bottom‐up synthesis methods are two main synthesis methods of 2D MOFs.^[^ ^14^ ^]^ The bottom‐up synthesis methods involve obtaining 2D nanosheets by breaking the interlayer interaction forces of the bulk MOF.^[^ ^15^ ^]^ The top‐down synthesis methods refer to the direct reaction of metal ions and organic linkers to selectively inhibit the growth of the 3D structure to obtain ultrathin nanosheets without affecting the 2D scale growth of the materials.^[^ ^16^ ^]^ 2D nanostructures are characterized by faster mass transport, lower diffusion barriers, and more exposed active sites, which allow molecules or ions to enter the active site directly. These properties make them ideal candidates for improving catalytic performance, for example, Zhao and co‐workers.^[^ ^17^ ^]^ constructed novel 2D Ni@Cu‐MOF nanosheets as high‐performance catalyst electrode materials for the electrooxidation of Glu in alkaline media. This Ni@Cu‐MOF sensor showed excellent electrochemical Glu sensing performance. Although a great deal of work has focused on 2D MOFs, little has been reported on the processes by which they are regulated.

Herein, we reported a regulated synthesis method of 2D lamellar Ni‐MOFs. In this method, the bidentate ligand 4,4′‐bipyridine (BPy) was used as an organic ligand and the monodentate ligand pyridine, which has a similar structure to BPy, was used as a regulator to selectively inhibit the growth of Ni‐MOF in the longitudinal dimension. By controlling the amount of regulator added, homogeneous lamellar Ni‐MOF was synthesized. In the 2D lamellar Ni‐MOF, PNMOF‐3 possessed the optimal Glu oxidation activity with a fast response time of <3 s, a high sensitivity of 907.54 µA mm ^−1^ cm^−2^, and a linear response width of 0.5–2665.5 µm. In addition, the sensor has good stability and can be cycled steadily for 4000 s. The sensor delivered good selectivity, good reproducibility, and may be used to detection Glu in real sample.

## 2 Results and Discussion

**Figure 1:**
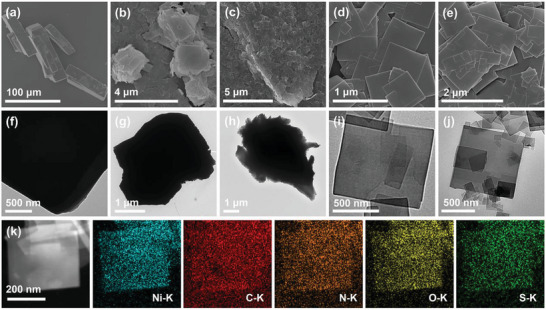
SEM images of a) PNMOF‐0; b) PNMOF‐1; c) PNMOF‐2; d) PNMOF‐3; e) PNMOF‐4, TEM images of f) PNMOF‐0; g) PNMOF‐1; h) PNMOF‐2; i) PNMOF‐3; j) PNMOF‐4, and k) EDS elemental mapping of prepared PNMOF‐3.

The reaction conditions for the conventional bulk Ni‐MOF were explored to some extent in order to obtain samples with homogeneous and regular shapes. Fixing the amount of NiSO_4_·6H_2_O and BPy in the reaction solution and the type and proportion of solvent, a series of green powders were obtained by varying the reaction times (1, 3, 6, 12, 24, 28 h) to investigate the growth process of bulk Ni‐MOF. The detailed molar quantities of each reactant and the names of each product were shown in Table S1 (Supporting Information). The bulk Ni‐MOF was donated as NMOF and the pydrine regulated lamellar Ni‐MOF was donated as PNMOF. From Figure S1 (Supporting Information) and **Figure** **1**a, it can be seen that at a reaction time of 1 h, the NMOF‐1 showed an irregular bulk structure. The bulk structure of NMOF‐2 with a lateral size of about 1 µm aggregated and self‐assembled into cube structure with the reaction time was extended to 3 h. By continuing to extend the reaction time to 6 h, the size of the sample continues to increase, the NMOF‐3 delivered a multilayer cubic structure. By the reaction time reaching 12 h, the bulk shape was essentially fixed, except for the poor homogeneity compared to the sample with a reaction time of 24 h. At 24 and 28 h, the shape of PNMOF‐0 and NMOF‐5 was essentially the same, with a homogeneous bulk structure. Therefore, a homogeneous bulk structure was obtained at a reaction time of 24 h, which was chosen as the reaction time for the subsequent reaction. Energy‐dispersive X‐ray spectrometry (EDS) elemental mapping revealed that characteristic elements of the bulk PNMOF‐0, such as Ni, C, N, O, and S, were homogeneously distributed throughout the material (Figure S1f, Supporting Information). Next, the reaction time was fixed and Ni‐MOFs were prepared by introducing pyridine as a regulator. The regulation process of the morphology of 2D Ni‐MOF was analyzed by scanning electron microscopy (SEM) images (Figure 1a–e), which showed that the bulk structure gradually disintegrated with the addition of pyridine, and the lateral size of PNMOF‐1 decreased from about 100 µm for PNMOF‐0 to about 3 µm when 0.5 mL of pyridine was added. When pyridine was added at 1.0 mL, lamellar structure began to appear, and the edges of the bulk structure became irregular. When 1.5 mL of pyridine was added, the PNMOF‐3 showed a regular lamellar structure with a homogeneous shape. However, when 2.0 mL of pyridine was added, the lamellar structure gradually disintegrated. From this, we can conclude that the regulator pyridine can effectively limit the growth of Ni‐MOF along the vertical direction and make the bulk Ni‐MOF downgraded to a uniform lamellar structure, and the uniform lamellar structure will also be destroyed when the addition of pyridine continued to increase. The transmission electron microscopy (TEM) images were in good agreement with SEM images (Figure 1f–j), and it was obvious that the thickness of the sample decreased significantly with the addition of pyridine. Figure 1k demonstrated that the uniformly shaped flakes of PNMOF‐3 possessed a uniform distribution of Ni, C, N, O, and S elements from SO_4_^2−^.

**Figure 2:**
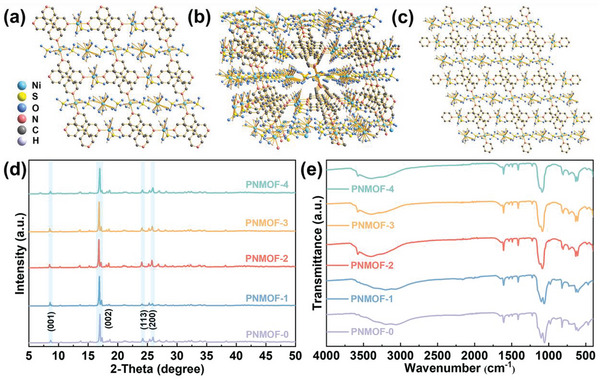
Structural analysis of Ni‐MOF: a) 2D stacking diagram of PNMOF‐0 along the *a*‐direction; b) 3D stacking diagram of PNMOF‐0 along the *c*‐direction; c) 2D stacking diagram of PNMOF‐3 along the *a*‐direction. d) Powder XRD patterns and e) FT‐IR spectra of PNMOF‐0, PNMOF‐1, PNMOF‐2, PNMOF‐3, PNMOF‐4.

In order to investigate the regulatory role of pyridine in the formation of the lamellar Ni‐MOF, the internal structure of the bulk and lamellar Ni‐MOF was studied in **Figure** **2**a–c. From Figure 2a, there were two different 1D chains in the *a*‐direction in the bulk PNMOF‐0, one with a Ni^2+^ ion linking 2 BPy, 2 SO_4_^2+^ ions, and 2 H_2_O molecules. The other was formed by a Ni^2+^ ion linked to two BPy, a SO_4_^2+^, and 3 H_2_O molecules. These two 1D chains were joined in a cross‐like pattern at the midpoint of the BPy and formed Ni(H_2_O)_6_^2+^ in the pore space. The stacking of these parallel aligned undulating 1D chains along the *c*‐axis results in a 3D supramolecular lattice structure, as shown in Figure 2b.^[^ ^18^ ^]^ Since the more basic the ligand (Lewis base) is for the same metal ion (Lewis acid), the greater the stability constant of the complex. Compared to the bidentate ligand BPy (p*K*_a1_ = 3.17; p*K*_a2_ = 4.82), the monodentate ligand pyridine has a stronger coordination ability (p*K*_a1_ = 5.25) and can therefore replace the original ligand for binding to the metal ion, while at the same time, a pyridine molecule has only one nitrogen atom available for coordination and can therefore act as a terminal capping ligand. When pyridine selectively engages in coordination in the vertical direction, the growth of the bulk structure along the vertical direction is inhibited and a lamellar structure is formed.^[^ ^19^ ^]^ From Figure 2c, it can be seen that the structure of the lamellar PNMOF‐3 in the *a*‐direction was essentially the same as that of PNMOF‐0, except that the addition of pyridine blocks the continued growth of the Ni‐MOF (pyridine was located at the end of the 2D stacking diagram). From the above analysis of the internal structure of Ni‐MOF, it is clear that the monodentate ligand pyridine selectively participated in the coordination to regulate the morphology of Ni‐MOF in the vertical direction by acting as a terminal capping ligand, and the coordination of pyridine will have some influence on the internal structure of Ni‐MOF, but since pyridine had the same elements and functional groups as BPy, its coordination did not introduce new functional groups.

The X‐ray diffraction (XRD) patterns showed that the five samples all have good crystallinity, which was indexed by the previously reported Ni‐MOF (CCDC No. 826371). In Figure 2d, the diffraction peaks at 2*θ* of 8.62°, 16.94°, 24.16°, and 25.88° were attributed to the (001), (002), (113), and (200) crystal planes of Ni‐MOF, respectively.^[^ ^6a^ ^]^ It should be noted that the diffraction peaks of PNMOF‐0, PNMOF‐1, PNMOF‐2, and PNMOF‐3 were essentially the same, and when the addition of pyridine was increased to 2.0 mL, a new diffraction peak appears at 6.86° for PNMOF‐4, indicating that the addition of pyridine inhibited the growth of the frame and induced the formation of a new phase and coordination structure.^[^ ^13^ ^]^ The Fourier transform infrared (FT‐IR) spectra in Figure 2e further exhibited that the five samples have similar absorption peaks, indicating that they shared the same surface groups and linkages, and the addition of the pyridine did not change the bonding pattern within the bulk PNMOF‐0, which was consistent with the internal analysis results of the Ni‐MOF. The absorption peaks of five samples at 1612, 1535, 1487, and 1409 cm^−1^ demonstrated that there was stretching vibrations of C═N and C═C in Ni‐MOF. The absorption peak of the samples at 1099 cm^−1^ confirmed the presence of SO_4_^2−^ and the absorption peak at 638 cm^−1^ originated from the Ni─O bond, indicating the presence of coordination bonds between Ni^2+^ and SO_4_^2−^.

**Figure 3:**
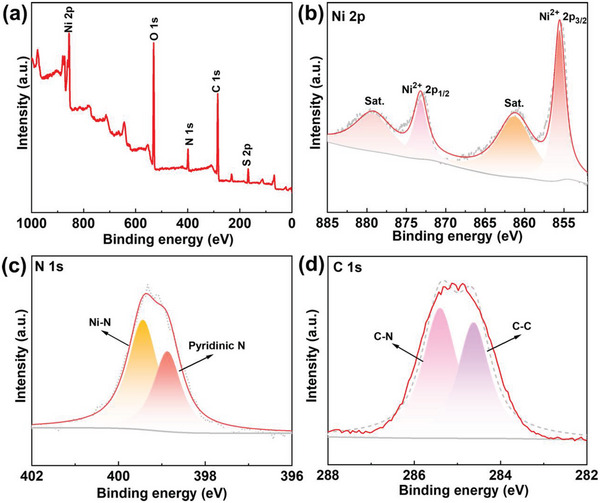
a) XPS survey spectra of PNMOF‐3; XPS spectra in the b) Co 2p, c) N 1s, and d) C 1s regions.

The types and valence states of the elements in the samples were determined by X‐ray photoelectron spectroscopy (XPS). From the survey spectra in **Figure** **3**a, the PNMOF‐3 contained Ni, C, N, O, and S elements, which was consistent with the results shown by EDS elemental mapping. In order to better identify the bonding valence states of the elements, the high‐resolution Ni 2p XPS spectra were analyzed (Figure 3b), and the peaks at 855.6 and 873.3 eV were attributed to Ni^2+^ 2p_3/2_ and Ni^2+^ 2p_1/2_, respectively, indicating that the metal ions were mainly present in the form of Ni^2+^ in PNMOF‐3.^[^ ^20^ ^]^ In the high‐resolution N 1s XPS spectra (Figure 3c), the peaks at 399.4 and 398.9 eV were attributed to the Ni─N bond and pyridine N, respectively, confirming the successful coordination of BPy and pyridine with Ni^2+^, which was consistent with the analysis of the internal structure mentioned above.^[^ ^21^ ^]^ The two peaks at 284.8 and 285.9 eV in the high‐resolution C 1s XPS spectra (Figure 3d), corresponded to the C─C and C─N, respectively.^[^ ^18^ ^]^

**Figure 4:**
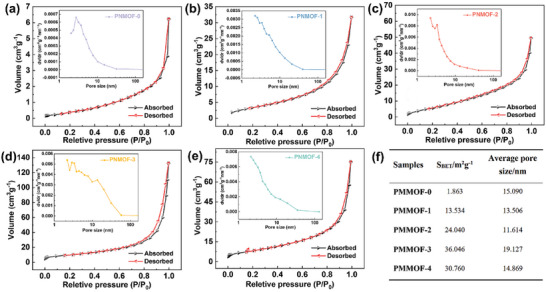
Nitrogen adsorption–desorption isotherms of a) PNMOF‐0; b) PNMOF‐1; c) PNMOF‐2; d) PNMOF‐3; e) PNMOF‐4 (inset: the corresponding pore size distribution at 77 K). f) The specific surface area and average pore diameter of the corresponding samples.

To investigate the effect of pyridine modulation on the specific surface area and pore structure of the samples, the N_2_ adsorption–desorption isotherms were shown in **Figure** **4**a–e, and their specific surface areas were calculated to be 1.863, 13.534, 24.040, 36.046, and 30.760 m^2^ g^−1^ (Figure 4f), respectively, indicating that the ratio of added pyridine has a large effect on the specific surface area of Ni‐MOF. Among all samples, PNMOF‐3 had the largest specific surface area, which can provide more active sites in electrocatalytic Glu sensing and increase the contact area between the substrate Glu and the active centers, thus improving the sensing performance. In addition, the pore size distribution curves indicated that all five samples were mesoporous in structure. The pore size of the bulk sample PNMOF‐0, which was mainly distributed around 3 nm, and the pore size of the PNMOF‐3 was relatively uniform in the range of 1–50 nm, indicating that the modulating effect of pyridine can make the material have a larger and more uniform pore size structure, which was conducive to the penetration of electrolyte and charge transfer, and can obtain more effective reactive active sites to accelerate the catalytic reaction.

To investigate the effect of bulk and lamellar structures on the electrocatalytic properties of the samples, cyclic voltammetry (CV) tests were carried out on PNMOF‐0 and PNMOF‐3 in 0.1 m NaOH with(out) 100 µm Glu at 50 mV s^−1^ over a voltage interval of 0–0.7 V. The solid line showed the results without the addition of Glu. For PNMOF‐3, the oxidation reaction of Ni^2+^ to Ni^3+^ occurred at around 0.5 V for the forward scan and the reduction reaction of Ni^3+^ to Ni^2+^ at around 0.4 V for the reverse scan (Figure S2a, Supporting Information). The dashed line shows the change in response current of PNMOF‐3 with the addition of 100 µm Glu. The response current was significantly higher than that without Glu, which may be due to the enhanced oxidation current value by the catalytic reaction of Ni^3+^ to Glu. The corresponding reaction mechanism in Ni‐MOF was transformed into Ni^3+^ by the electric field, and part of Ni^3+^ catalyzes the oxidation reaction of Glu to gluconolactone under alkaline conditions, which itself was reduced to Ni^2+^, resulting in an increase in the oxidation current, and the corresponding reaction mechanisms were shown in the following Equations (1) and (2)

(1) Ni(II)−MOF→Ni(III)−MOF+e−

(2) $$\begin{array}{ccc} & & \mathrm{Ni}(\mathrm{III})-\mathrm{MOF}\;+\;\mathrm{OH}-\;+\;\mathrm{Glu}\;\to \;\mathrm{Ni}(\mathrm{II})-\mathrm{MOF}\\ & & \;+\;\mathrm{Glucolactone}\;+\;H2O\;+\;e- \end{array}$$

Similarly, an increase in the concentration of Glu in 0.1 m NaOH also led to an enhancement of the current response of PNMOF‐0 glass carbon electrode (GCE), indicating that PNMOF‐0 can also catalyze the oxidation of Glu. Compared to the lamellar PNMOF‐3, the current response signal of PNMOF‐0 was slightly weaker. Combined with the morphological changes of the material, it was speculated that with the addition of pyridine, the bulk structure gradually disintegrates to form a regular and homogeneous lamellar structure with more nanopores distributed on the surface of these lamellar structures, making the specific surface area of PNMOF‐3 larger, which was conducive to the contact between the Glu and the active centers, while the porous structure facilitated the adsorption and desorption of the reactants, electron transfer and increased the reaction rate, thus the response current was significantly enhanced when Glu was added. Figure S2b (Supporting Information) showed the comparison of the intensity of the response currents of PNMOF‐0 and PNMOF‐3 GCE when different amounts of Glu were added successively, which can further confirm the conclusion that the PNMOF‐3 had a stronger response current. It can also be seen that the lamellar structure was more sensitive to the Glu catalytic response compared to the bulk structure and was more suitable for the preparation of nonenzymatic electrochemical Glu sensors. The next discussion will mainly focus on the pyridine‐regulated lamellar Ni‐MOF.

**Figure 5:**
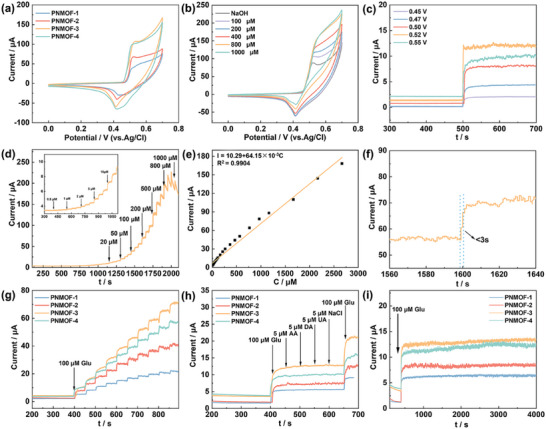
a) CV curves of the PNMOF‐1, PNMOF‐2, PNMOF‐3, PNMOF‐4 GCE in 0.1 m NaOH with 10 µm Glu; b) CV curves of PNMOF‐3 GCE in 0.1 m NaOH when adding different concentrations of Glu; c) amperometric responses of the PNMOF‐3 GCE at different potentials (from 0.40 to 0.55 V) with continuous addition of 100 µm Glu in 0.1 m NaOH; d) *i*–*t* response of the PNMOF‐3 GCE at 0.55 V on successive additions of different amounts of Glu in 0.1 m NaOH; e) calibration curve of PNMOF‐3 GCE for current response to Glu concentration in the range of 0.5 µm–3.0 mm; f) response time for PNMOF‐3 GCE after the addition Glu solution; g) *i*–*t* response of the PNMOF‐1, PNMOF‐2, PNMOF‐3, PNMOF‐4 GCE with the continuous addition of 100 µm Glu at 0.55 V; h) *i*–*t* responses of the PNMOF‐1, PNMOF‐2, PNMOF‐3, PNMOF‐4 GCE with the addition of 100 µm Glu, 5 µm ascorbic acid (AA), 5 µm dopamine (DA), 5 µm urea (UA), 5 µm NaCl, and 100 µm Glu into 0.1 m NaOH at 0.55 V; i) the stability of the response current for the PNMOF‐1, PNMOF‐2, PNMOF‐3, PNMOF‐4 GCE after the addition of Glu solution (100 µm) over 4000 s.

The comparative CV curves of PNMOF‐1, PNMOF‐2, PNMOF‐3, PNMOF‐4 GCE in 0.1 m NaOH (100 µm Glu) were shown in **Figure** **5**a. As the amount of pyridine added increased from 0.5, 1.0, and 1.5 mL, the samples gradually disintegrated from irregular lumps to homogeneous flakes, and the increase of specific surface area was conducive to the improvement of catalytic activity of the material, so the response current gradually increased. When pyridine was added at 2.0 mL, the lamellar structure gradually disintegrated and the specific surface area of PNMOF‐4 decreased, so the response current decreased compared to that of the PNMOF‐3 GCE. In combination with the above analysis, it is clear that the PNMOF‐3 GCE had the advantage of greater response current among these materials and was more suitable for the construction of nonenzymatic electrochemical Glu sensors. Figure 5b shows the CV curves of PNMOF‐3 GCE in different concentrations of Glu solution, which showed that the response current intensity of PNMOF‐3 GCE gradually increases with the increase of Glu addition, which indicated that PNMOF‐3 GCE had good catalytic ability for Glu in a wide range of concentrations and superior potential for constructing Glu sensors. The same CV tests were carried out for PNMOF‐1, PNMOF‐2, and PNMOF‐4 GCE and the results were shown in Figure S3 (Supporting Information). The response currents of the electrodes increased significantly with the increase of Glu addition, indicating that the PNMOF‐1, PNMOF‐2, and PNMOF‐4 GCE all had excellent electrocatalytic ability for Glu, and the comparison of the response current intensities when the same amount of Glu was added indicated that the lamellar structure with a larger specific surface area had better electrochemical performance than the bulk or bulk and lamellar coexisting structures.

In order to obtain the best amperometric signal for PNMOF‐3, the modified electrode was tested at an applied potential of 0.45–0.55 with a stepwise addition of Glu (100 µm) (Figure 5c). At all five sets of voltages, the PNMOF‐3 GCE was able to achieve the catalytic response to Glu. As the voltage increased from 0.45 to 0.52 V, the response current gradually increased, and when the test voltage continued to increase to 0.55 V, the response current decreased slightly compared to 0.52 V. Therefore, 0.52 V, which had the largest response value and relatively smooth curve, was selected as the operating voltage for the following tests.

The current–time (*i*–*t*) curve for successive additions of a certain amount of Glu at 50 s intervals was shown in Figure 5d, which delivered that the current value jumped rapidly and reached a plateau with the addition of Glu, which indicated that PNMOF‐3 GCE can achieve a fast and stable response to Glu. From Figure 5d inset, it can be seen that even a low addition of Glu can cause a significant current response, indicating that the PNMOF‐3 GCE can achieve a sensitive detection of Glu even at a low concentration. Figure 5e showed a linear fit of the Glu concentration to the response current intensity in the *i*–*t* curve, which provided a more visual representation of the linear relationship between the two. The linear fit equation was *I* (µA) = 10.29 + 64.15 × 10^−3^ C (µm) with *R* ^2^ = 0.9904 for Glu concentrations between 0.5 and 2665.5 µm, giving a sensitivity of 907.54 µA mm ^−1^ cm^−2^. In addition to the linear detection interval and sensitivity, the response time of the electrode to Glu is also an important factor in evaluating the performance of the electrode. Figure S4a–c (Supporting Information) shows the *i*–*t* curves of PNMOF‐1, PNMOF‐2, PNMOF‐4 GCE when different amounts of Glu were added continuously. Each electrode showed an obvious and stable current response to Glu, and the larger the amount of Glu added, the more obvious the current response was, and the overall trend of the curve was a step‐up, indicating that PNMOF‐1, PNMOF‐2, PNMOF‐4 GCE all had good electrocatalytic ability to Glu. By linearly fitting the data, as shown in Figure S4d–f (Supporting Information), it was found that the current response signals of PNMOF‐1, PNMOF‐2, PNMOF‐4 GCE showed excellent linearity with a wide range of linearity with respect to Glu concentration. As shown in Figure S4d (Supporting Information), the sensitivity of PNMOF‐1 GCE could reach 536.03 µA mm ^−1^ cm^−2^ when the Glu concentration ranged from 0.5 to 1165.5 µm. Figure S4e (Supporting Information) showed that the sensitivity of PNMOF‐2 GCE could reach 674.96 µA mm ^−1^ cm^−2^ when the Glu concentration ranged from 0.5 to 2165.5 µm. Figure S4f (Supporting Information) showed that the sensitivity of PNMOF‐4 GCE was 846.98 µA mm ^−1^ cm^−2^ for Glu concentrations between 0.5 and 2665.5 µm. By comparing the above data with the electrochemical data of PNMOF‐3 GCE, it was concluded that the electrode prepared from the PNMOF‐3 sample had a more uniform shape and a larger specific surface area, with a wider detection interval and higher sensitivity. In addition to the linear detection interval and sensitivity, the response time of the electrode to Glu was also an important parameter for evaluating the performance of the electrode. As shown in Figure 5f, when 200 µm Glu was added to the electrolyte, the PNMOF‐3 GCE could achieve a fast response within 3 s and had the ability to construct an instant Glu concentration detection sensor.

To avoid experimental chance, a repeatability test was performed on PNMOF‐3 GCE. As shown in Figure S5a (Supporting Information), the current response was similar for 10 repetitions of the addition of 100 µm Glu, from Equation (3)

(3) $$RSD=\frac{S}{x}\times 100\%=\frac{\frac{\sum_{i=1}^{n}{\left(x_{i}\;-\;\bar{x}\right)}^{2}}{n\;-\;1}}{\bar{x}}\times 100\%$$

The relative standard deviation (RSD) of the response currents was calculated to be 4.24%, indicating a good repeatability of the response of PNMOF‐3 GCE to Glu (*S*: standard deviation; $\bar{x}$ mean). In order to test the confidence of the PNMOF‐3 GCE detection results, Glu and various interferents were added sequentially to test the anti‐interference ability of the electrode under the premise that the *i*–*t* curve reached stability. As shown in Figure S5b (Supporting Information), the addition of interferences did not produce significant fluctuations in the response current, so it can be concluded that PNMOF‐3 GCE had no current response to the above interferents and showed good immunity to interference. Furthermore, in the presence of multiple interferents, the PNMOF‐3 GCE still produced a very strong electrical signal to Glu with no attenuation in response strength, proving its superior commercial potential. In the stability test in Figure S5c (Supporting Information), the current response of PNMOF‐3 GCE was always smooth during the test up to 4000 s, indicating that PNMOF‐3 GCE exhibited good electrochemical stability and can meet the requirements of long‐time testing.

Figure 5g delivered the good repeatability of the response currents for all four electrodes when testing the same amount of Glu over 10 repetitions, with the PNMOF‐3 GCE showing the greatest current response. Figure 5h showed that all four electrodes have no significant current response to the interferents: ascorbic acid, dopamine, uric acid, and sodium chloride, and have good immunity to interference. Also, in the presence of the above interferents, the electrodes show no significant attenuation of the response to 100 µm Glu and have the ability to accurately detect Glu concentrations in complex environments. Figure 5i showed that all four electrodes have good electrochemical stability and the response current remains smooth throughout the 4000 s electrochemical test, enabling the detection of Glu concentrations over a long period of time. Compared to PNMOF‐1, PNMOF‐2, and PNMOF‐4, PNMOF‐3 GCE showed the strongest response current in the tests of repeatability, immunity, and stability, which was more useful for sensitive detection of Glu. As shown in Table S2 (Supporting Information), when comparing the electrochemical performance with other reported electrodes, it can be found that the PNMOF‐3 GCE was more sensitive over a wider detection range and therefore, it is considered that the PNMOF‐3 GCE prepared by this pyridine‐regulated method had good electrochemical Glu sensing performance.

## 3 Conclusion

Five Ni‐MOFs with different morphologies were synthesized by a simple one‐step hydrothermal using NiSO_4_·6H_2_O as the metal source and BPy as the organic ligand, and controlling the addition of the pyridine as regulator. During the growth of the Ni‐MOF, the bidentate organic ligand BPy with Ni^2+^ makes the materials grow in 3D direction, while the monodentate ligand pyridine, which has a similar structure to BPy, can compete with it for coordination and selectively inhibit the growth of Ni‐MOF in the longitudinal dimension to synthesize lamellar structures, by controlling the addition of the pyridine regulator to regulate the morphological structure of the Ni‐MOF. As we envisaged, PNMOF‐0 possessed the bulk structure iwithout the addition of pyridine, and the bulk structure gradually disintegrated with increasing pyridine addition (PNMOF‐1, PNMOF‐2), until a more homogeneous lamellar PNMOF‐3 was obtained at 1.5 mL of pyridine addition, after which the lamellar structure was found to be gradually destroyed when the pyridine addition was increased to 2.0 mL (PNMOF‐4), confirming the effect of the regulator pyridine on the degradation effect of pyridine on the bulk MOF. Characterization of the five structures revealed that they had similar crystal structures and functional groups, and the change in morphology from bulk to lamellar structure increased the specific surface area, providing more active sites for the catalytic reaction of Glu. In the electrochemical performance comparison, it was found that the more homogeneous morphology and larger specific surface area of the lamellar PNMOF‐3 exhibited the highest Glu electrocatalytic activity, with a sensitivity of 907.54 µA mm ^−1^ cm^−2^ at Glu concentrations between 0.5 and 2665.5 µm, a significant improvement in electrochemical performance compared to the pristine bulk PNMOF‐0.

## Conflict of Interest

The authors declare no conflict of interest.

## Supporting information

Supporting Information

## Data Availability

Research data are not shared.
