# Supplementary material for: Pyridine‐Regulated Lamellar Nickel‐Based Metal–Organic Framework (Ni‐MOF) for Nonenzymatic Electrochemical Glucose Sensor
Source: Adv Sci (Weinh). 2023 Jul 20;10(27):2304102. doi: 10.1002/advs.202304102 (PMC10520646; doi:10.1002/advs.202304102)
Supplement: Supplementary file 1 — Supporting Information [file ADVS-10-2304102-s001.pdf]

ADVANCED SCIENCE
Open Access

Supporting Information

for *Adv. Sci.*, DOI 10.1002/advs.202304102

Pyridine-Regulated Lamellar Nickel-Based Metal–Organic Framework (Ni-MOF) for Nonenzymatic Electrochemical Glucose Sensor

Qian Zhang, Panpan Li, Jun Wu, Yi Peng and Huan Pang**

Supporting Information

Pyridine-regulated lamellar nickel-based metal organic framework (Ni-MOF) for non-enzymatic electrochemical glucose sensor

Qian Zhang, Panpan Li, Jun Wu, Yi Peng, Huan Pang**

Q. Zhang, P. Li, J. Wu, Y. Peng, H. Pang
School of Chemistry and Chemical Engineering
Yangzhou University
Yangzhou 225009, P. R. China
* E-mail: panghuan@yzu.edu.cn (H. Pang), huanpangchem@hotmail.com (H. Pang)
yipengchem@hotmail.com (Y. Peng)
Homepage: https://www.x-mol.com/groups/panghuan

Materials

All chemicals, including $NiSO_4 \cdot 6H_2O$, 4,4′-bipyridine (BPy), pyridine, ethanol (EtOH), were purchased from Shanghai Sinopharm Chemical Reagent and used without further treatment or purification. Nafion solution (5 wt%) was purchased from Aladdin Reagent (Shanghai) Company. All aqueous solutions were prepared with deionized water (resistance 18 MU cm^{-1})

Experimental Section

Preparation of bulk Ni-MOF

0.52 g $NiSO_4 \cdot 6H_2O$, 0.31 g BPy were dissolved in a mixed solution consisting of 15 mL deionized water and 7.5 mL EtOH and stirred at room temperature for 10 min, the above solution was transferred to a 50 mL Teflon-lined stainless autoclave and the reaction was carried out at 100 °C. The products with reaction time of 1 h, 3 h, 6 h, 12 h, 24 h and 28 h were prepared respectively. After cooling down to the room temperature, solid products were collected by centrifugation. The solid was then washed with EtOH (10 mL × 3) and deionized water (10 mL × 3) dried at 50 °C under vacuum.

Preparation of 2D Ni-MOF nanosheets

0.52 g $NiSO_4 \cdot 6H_2O$, 0.31 g BPy were dissolved in 15 mL of deionized water and stirred at room temperature for 10 min. Four of the above aqueous solutions were prepared. Different amounts (0.5 mL, 1 mL, 1.5 mL and 2 mL) of pyridine were dissolved in 7.5 mL of EtOH, respectively. The above solution was added drop by drop to the aqueous solution separately and stirred at room temperature for the above mixtures were transferred to 50 mL Teflon-lined stainless autoclaves and the reaction was carried out at 100 °C for 24 h. After cooling down to the room temperature, solid products were collected by centrifugation. The solid was then washed with EtOH (10 mL × 3) and deionized water (10 mL × 3) dried at 50 °C under vacuum.

Characterization

The morphological features were characterized by field emission scanning electron microscopy (FESEM, Zeiss-Supra55), high resolution transmission electron microscopy (HRTEM, Tecnai G2 F30 S-TWIN), and energy dispersive X-ray spectrometry (EDS) mapping. X-ray diffraction (XRD) patterns were examined on a Bruker D8 Advanced X- ray Diffractometer (Cu-Kα radiation: λ = 0.15406 nm). The chemical states were measured using an Axis Ultra X-ray photoelectron spectroscope (XPS, Kratos Analytical Ltd., UK) equipped with a standard monochromatic Al-Kα source (hv = 1486.6 eV). Fourier transform infrared (FTIR) transmission spectra were obtained on a BRUKER-EQUINOX-55 IR spectrophotometer. N_2 adsorption-desorption measurements were performed on Quantachrome Instruments, Autosorb IQ3.

Fabrication of working electrodes and electrochemical measurements

To fabricate the working electrodes, a mixture containing of the as-synthesized MOFs (3 mg), 5 wt% Nafion solution 25 μL, and 300 μL of water, 250 μL of EtOH was well mixed. Then the solution was ultrasonicated for one hour. 5 μL mixture was dripped on the surface of glassy carbon electrode and dried for two hours at room temperature.

Electrochemical measurements were conducted on a CHI660e electrochemical station (CH Instruments, Shanghai, China). A conventional three-electrode system was used for all electrochemical measurements, which consisted of a modified GCE electrode as the working electrode, an Ag/AgCl (saturated KCl) electrode as the reference electrode, and platinum foil as the auxiliary electrode in 0.1 M NaOH with the protection of N_2.

Table S1. Molar dosages of the reactants in hydrothermal reactions.

	$NiSO_4{\cdot}6H_2O$	BPy	H_2O	EtOH	Pyridine	Reaction Time
**NMOF-1**	0.52 g	0.31 g	15 mL	7.5 mL	0 mL	1 h
**NMOF-2**	0.52 g	0.31 g	15 mL	7.5 mL	0 mL	3 h
**NMOF-3**	0.52 g	0.31 g	15 mL	7.5 mL	0 mL	6 h
**NMOF-4**	0.52 g	0.31 g	15 mL	7.5 mL	0 mL	12 h
**PNMOF-0**	0.52 g	0.31 g	15 mL	7.5 mL	0 mL	24 h
**PNMOF-1**	0.52 g	0.31 g	15 mL	7.5 mL	0.5 mL	24 h
**PNMOF-2**	0.52 g	0.31 g	15 mL	7.5 mL	1 mL	24 h
**PNMOF-3**	0.52 g	0.31 g	15 mL	7.5 mL	1.5 mL	24 h
**PNMOF-4**	0.52 g	0.31 g	15 mL	7.5 mL	2 mL	24 h
**NMOF-5**	0.52 g	0.31 g	15 mL	7.5 mL	0 mL	28 h

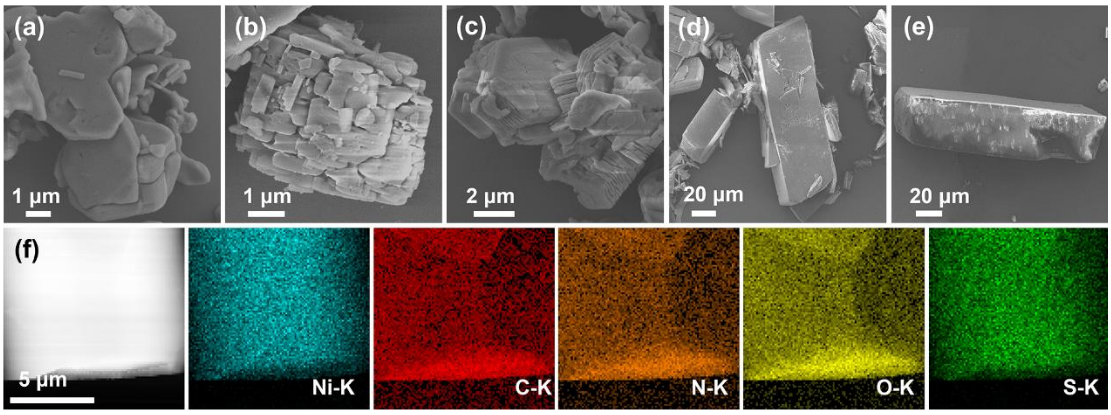

Figure S1. SEM images of (a) NMOF-1; (b) NMOF-2; (c) NMOF-3; (d) NMOF-4; (e)NMOF-5, (f) HAADF-STEM images and elemental mappings of Ni-K, Co-K, C-K, N-K, O-K, S-K, and in PNMOF-0.

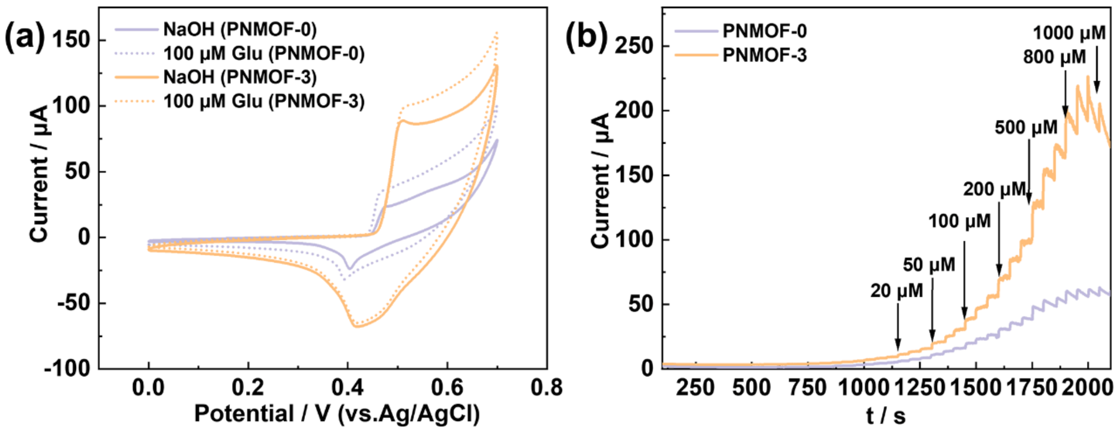

Figure S2. (a) CV curves of PNMOF-0 and PNMOF-3 electrodes in 0.1 M NaOH solution with and without 100 μM Glu; (b) Corresponding i-t curves during successive dropwise addition of different concentrations of Glu.

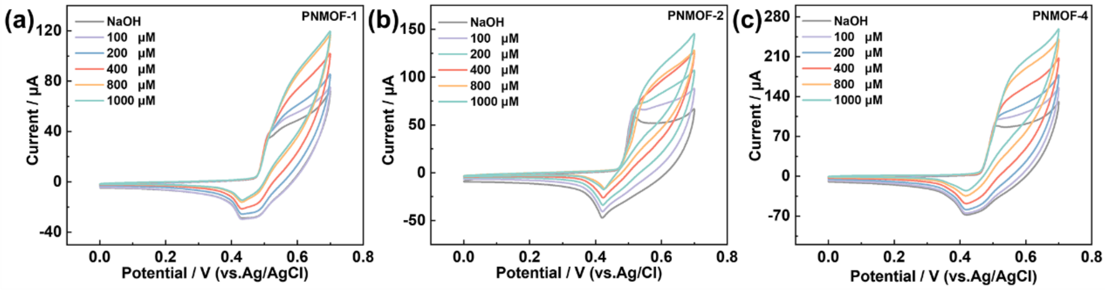

Figure S3. CV curves of (a)PNMOF-1; (b)PNMOF-2; (c) PNMOF-3 in 0.1 M NaOH when adding different concentrations of Glu.

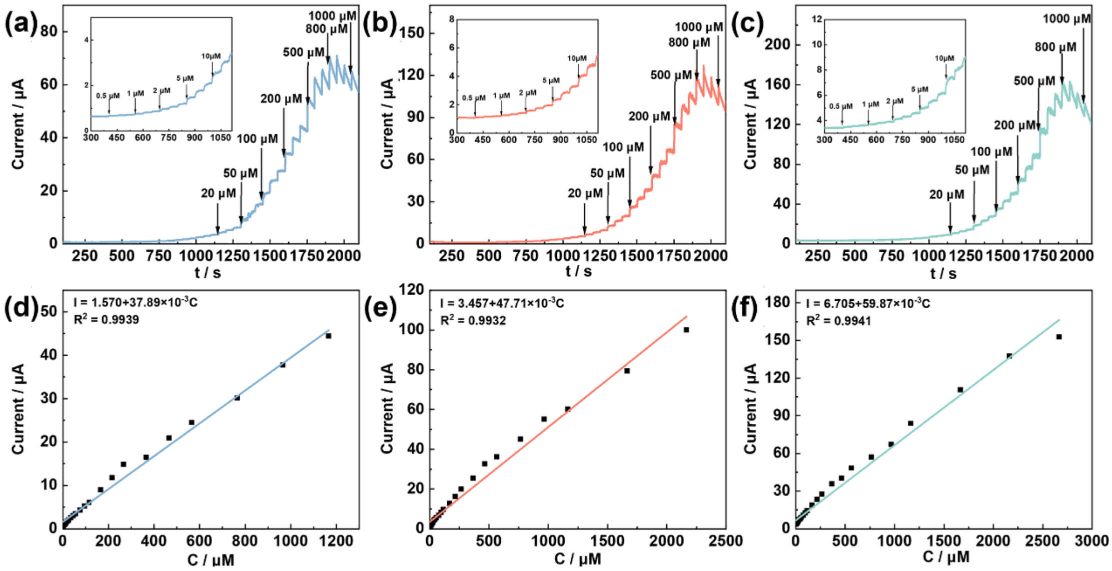

Figure S4. i-t response of the (a) PNMOF-1; (b) PNMOF-2; (c)PNMOF-3 GCE at 0.55 V on successive additions of different amounts of Glu in 0.1 M NaOH. Calibration curve of (d) PNMOF-1; (e) PNMOF-2; (f)PNMOF-4 GCE for current response to different Glu concentration.

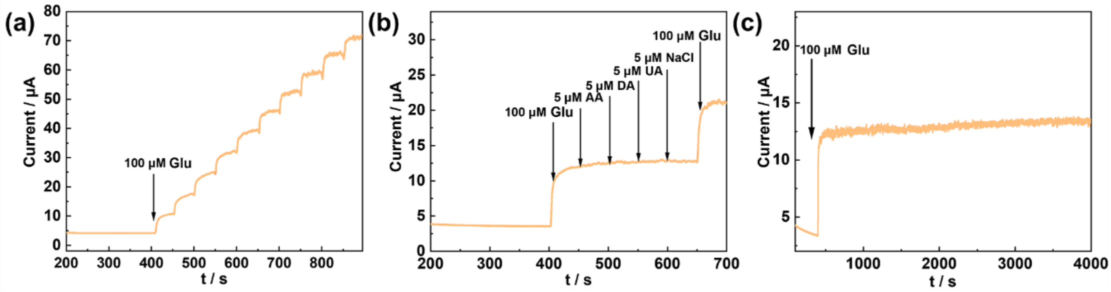

Figure S5. Electrochemical performances of the PNMOF-3 GCE: (a) i-t response with the continuous addition of 100 μM Glu at 0.55 V; (b) i-t response with the addition of 100 μM Glu, 5 μM AA, 5 μM DA, 5 μM UA, 5 μM NaCl, and 100 μM Glu into 0.1 M NaOH at 0.55 V; (c) The stability of the response current after the addition of Glu solution (100 μM) over 4000 s.

Table S2. Comparison of non-enzymatic electrochemical glucose sensor performance of PNMOF-3 GCE with other reported electrodes

Catalysts	low detection limit (μM)	Linear range (μM)	Response sensitivity ($\mu A\ mM^{-1}\ cm^{-2}$)	Ref.
MOF-$Ni(OH)_2/TiO_2$	8	30 to 1,4000	192	[1]
Ni-MIL-77(MOF)	0.25	1 to 500	1.542	[2]
Nano NiO	0.16	1 to 110	55.9	[3]
MOF-NiO/Foam Ni	6.15	18 to 1200	395	[4]
Ni-rGO	0.55	1 to 110	813	[5]
Au-CeO_2	10	Up to 10,000	57.5	[6]
MOF-Co_3O_4	0.1	1 to 300	471.5	[7]
Ag@Ni-MOF	5	5 to 500	168.8	[8]
Ni-MOF/RGO/CF	0.6	6 to 2090	852	[9]
Ni-MOF	0.76	1 to 1600	2859.95	[10]
PNMOF-1	1.32	0.5 to 1165.5	536.03	This work
PNMOF-2	0.93	0.5 to 2165.5	674.96	This work
PNMOF-3	1.64	0.5 to 2665.5	907.54	This work
PNMOF-4	0.42	0.5 to 2665.5	846.98	This work

Reference

[1] A. Gao, X.-M. Zhang, X. Peng, H. Yu, L. Bai, W.-H. Jin, G.-S. Wu, R.-Q. Hang, P.-K. Chu, Sens. and Actuators B: Chem., 232 (2016) 150-157.

[2] X. Xiao, S.-S. Zheng, X.-R. Li, G.-X. Zhang, X.-T. Guo, H.-G. Xue, H. Pang, J. Mater. Chem. B, 5 (2017) 5234-5239.

[3] Y. Mu, D.-L Jia, Y.-Y. He, Y.-Q. Mao, H.-L. Wu, Biosens. Bioelectron., 26 (2011) 2948-2952.

[4] L. Wang, Y.-Z. Xie, C.-T. Wei, X.-P. Lu, X. Li, Y.-H. Song, Electrochim. Acta, 174 (2015) 846-852.

[5] Z.-G. Wang, Y. Hu, W.L. Yang, M.-J. Zhou, X. Hu, Sensors, 12 (2012) 4860-4869.

[6] M. Gougis, A. Pereira, D.-L. Ma, M. Mohamedi, RSC Adv., 4 (2014) 39955-39961.

[7] L. Han, D.-P. Yang, A.-H. Liu, Biosens. Bioelectron., 63 (2015) 145-152.

[8] J.-Z. Cao, J.-H. Yun, N.-H. Zhang, Y.-M. Wei, H. Yang, Z.-L Xu, Synthetic Met., 282 (2021) 116931.

[9] S. Dong, H.-W Niu, L.-W Sun, S.-X Zhang, D.-Q Wu, Z. Yang, M. Xiang, J Electroanal. Chem., 911 (2022) 116219.

[10] M. Zeraati, V. Alizadeh, P. Kazemzadeh, M. Safinejad, H. Kazemian, G. Sargazi, J. Porous Mater., 29,(2022) 257-267.
